# Determination of formaldehyde in textile dye and auxiliary chemicals with headspace gas chromatography-flame ionization detector

**DOI:** 10.55730/1300-0527.3330

**Published:** 2021-12-31

**Authors:** Korel GÜNEŞ, Ziya CAN, Ayşem ARDA

**Affiliations:** 1 Department of Chemistry, Faculty of Engineering, İstanbul University - Cerrahpaşa, İstanbul, Turkey; 2 Department of Chemistry, Institute of Graduate Studies, İstanbul University – Cerrahpaşa, İstanbul, Turkey

**Keywords:** Formaldehyde, textile dye, derivatization, gas chromatography, flame ionization detector, textile auxiliary chemicals

## Abstract

Formaldehyde is a chemical used in many different industrial sectors and is classified as a carcinogen as well as causing allergic reactions. Due to the health effects of formaldehyde, which is widely used in textile dyes and auxiliary chemicals, its analysis in this type of matrices becomes important. In this study, in order to meet these requirements, formaldehyde was converted to oxime derivative by derivatization with o-(2,3,4,5,6-Pentafluorobenzyl)hydroxylamine hydrochloride. This derivative was successfully sampled with the headspace technique and analyzed with a gas chromatography-flame ionization detector (GC-FID). For the developed method, derivatization and salt effect parameters were investigated. For the method, the limit of detection (LOD) was found as 0.05 mg L^−1^ and real sample analyzes were made for different types of dyes used in the textile industry, and sodium naphthalene sulfoxylate formaldehyde. In addition, the developed method was validated with sodium naphthalene sulfoxylate formaldehyde against ISO 14184-1 method by applying student t-, F-tests and no difference was found in terms of accuracy and precision. Thus, the applicability of the developed method to dyes and auxiliary chemicals used in the textile industry was successfully demonstrated. This allows all quality control processes to be handled using only one method.

## 1. Introduction

Formaldehyde has been used in many different areas, like the production of wood products such as medium-density fiberboard (MDF) and chipboard, which are mainly used in the construction sector, textile industry [1], cosmetics [2], household cleaning products, and the automotive industry [3]. Also, it is mostly used in the production of urea-formaldehyde, phenol-formaldehyde, and melamine-formaldehyde resins. Since the late 1920s, melamine-formaldehyde resins have been used as a cross-linker in textile products to prevent wrinkles [4], and as a binder in dyeing processes with disperse and pigment dyes [5]. The International Agency for Research on Cancer declared in 2004 that formaldehyde is classified as a carcinogen [6] and it was known that from the literature it causes allergic contact dermatitis in case of contact with the skin at sufficient concentration [7]. Therefore, the analysis of formaldehyde and formaldehyde-containing chemicals has become increasingly important. In addition, to perform the analysis successfully, several sampling techniques for different matrix environments were developed. According to this, in the literature mainly spectrophotometric [8–14], spectrofluorometric [6, 15–18], high-performance liquid chromatography (HPLC) [19–23], and gas chromatography (GC) [2, 24–26] methods were developed. Also, alternative methods were developed for the detection of formaldehyde such as biosensors [27], interdigitated microelectrode array [28], flow-injection chemiluminescence [29], and mobile phone-based [30]. In quality control laboratories, formaldehyde analyzes are usually carried out with acetylacetone-based Nash reagent [31], which is an International Organization for Standardization method (ISO 14184-1) [32]. As the researches on the use of formaldehyde and its effects on health increases, limitations in legal regulations increase. As a result, methods with low detection limits and fast results with practical pretreatments are needed. Headspace is a sampling method used in gas chromatography and has many advantages. With this method, qualitative and quantitative analyzes of analytes in solid and liquid samples can be performed with great precision. In addition, complex matrices that should not be injected into the gas chromatography can be easily analyzed without the need for additional processing and chemicals, thanks to this technique. Aqueous samples, which have a disruptive effect on most analytical gas chromatography columns, can also be analyzed without using an additional extraction procedure. Also, analytes can be converted into suitable derivatives using various derivatizing agents in order to be analyzed by gas chromatography.

In the developed formaldehyde analysis method, o-(2,3,4,5,6-Pentafluorobenzyl)hydroxylamine hydrochloride (PFBHA), which is widely used for derivatization of formaldehyde in the literature [33–35], was used together with the headspace technique. The oxime derivative formed was analyzed by GC-FID. For method optimization, derivatization time and temperature, PFBHA concentration, salt effect, and salt concentration were investigated. Real sample analyses were performed for disperse dyes, fluorescent pigment dyes, and sodium naphthalene sulfoxylate formaldehyde. Finally, the developed method was validated against the ISO 14184-1 method with the aid of the student t-test and F test.

## 2. Materials and methods

### 2.1. Instrumentation and chemicals

Precisa LS 220A SCS (Dietikon, Switzerland) analytical balance was used for weighing. GC-FID analysis was performed with Shimadzu GC-2010 Plus (Kyoto, Japon) equipped with Shimadzu AOC-5000 Plus (Kyoto, Japon) autosampler and Restek Rtx-Wax capillary column (30 m × 0.32 mm ID, 0.25 μm film thickness) (Pennsylvania, United States). For absorption measurements of the validation method, Shimadzu UV-1800 (Kyoto, Japon) spectrophotometer was used.

Formaldehyde standard, o-(2,3,4,5,6-Pentafluorobenzyl)hydroxylamine hydrochloride as derivatization reagent, 1-bromo-4-fluorobenzene (BFB) as internal standard were purchased from Sigma-Aldrich Chemie GmbH (Steinheim, Germany). Sodium chloride, potassium chloride, calcium chloride dihydrate, acetylacetone, acetic acid (glacial) were provided from Merck KGaA (Darmstadt, Germany).

### 2.2. Preparation of solution

PFBHA solution (1000 mg L^−1^), BFB solution (100 mg L^−1^), and stock formaldehyde solution (1000 mg L^−1^) were prepared in ultrapure water. Also, for citrate-buffer solutions trisodium citrate (0.1 mol L^−1^) and citric acid (0.1 mol L^−1^) stocks were prepared in ultrapure water and mixed appropriate volumes.

For the preparation of the Nash reagent, 150 g ammonium acetate was dissolved with 800 mL ultrapure water. After that, 3 mL of glacial acetic acid and 2 mL of acetylacetone were added to this solution. Finally, this mixture was diluted to 1000 mL.

### 2.3. Headspace and GC-FID conditions

For the headspace module, 2 mL of PFBHA solution and 0.5 mL of formaldehyde solution were mixed in a 20 mL headspace vial. The incubation temperature was 60 °C and the incubation time was 30 min. The injector temperature was 150 °C and the stirring rate was 500 rpm. The split injection was performed with a split ratio of 10 and injection volume of 1000 μL. The injection temperature was 300 °C. For the temperature program, the starting temperature of the oven was set to 60 °C. In the first step, the temperature was increased at a rate of 5 °C min^−1^ to the temperature of 120 °C. In the second step, the temperature was increased at a rate of 20 °C min^−1^ to the final temperature of 200 °C which was held for 5 min. The detector temperature was 300 °C. The carrier gas was nitrogen, and its flow rate was 5.56 mL min^−1^.

### 2.4. Real samples analysis

For the real sample analysis with the developed method, samples were selected from used extensively in the textile sector which has a risk of formaldehyde contain. Disperse dye (1 g), 1 g of fluorescent pigment dye, and 0.5 g of sodium naphthalene sulfoxylate formaldehyde were dispersed in 100 mL of ultrapure water and the developed method was performed.

### 2.5. Statistical analysis

To compare the developed method ISO 14184-1 method, which is widely used in textile laboratories, was used. The basis of the method is the measurement of the absorbance at 412 nm of the yellow-colored product formed by derivatizing formaldehyde with Nash reagent. Due to the color of the dyes used in real sample analysis interfere the method, only sodium naphthalene formaldehyde sulfoxylate could be analyzed. Sample (5 mL)and Nash reagent (5 mL) were added into the amber tubes and they were kept by shaking for 30 min at 40 °C. Finally, the solution was kept at room conditions for 30 min to come to room temperature. Absorbance measurements were carried out against the reagent blank. The obtained results from GC-FID and spectrophotometric methods were used for statistical comparison using the student t-test and F test.

## 3. Results

### 3.1. Derivatization of formaldehyde

**Figure 1:**
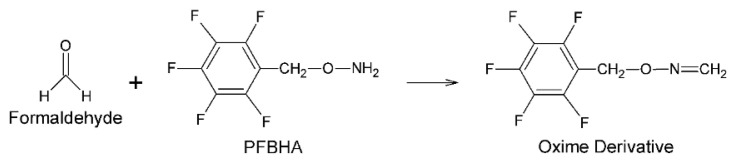
Oxime derivative formed by the reaction between formaldehyde and PFBHA.

As known from the literature, nucleophilic addition, protonation, and water elimination reactions take place between formaldehyde and PFBHA, respectively [35]. Thus, it is converted to the detectable oxime derivative (Figure 1). Formaldehyde-oxime formed can be detected in FID detectors thanks to its high carbon number, and in MS detectors due to its high molecular weight and functional groups that can be detached from the structure. It is also suitable for the headspace sampling technique due to the high vapor pressure.

### 3.2. Optimization of reaction parameters and analytical figures of merits

**Figure 2:**
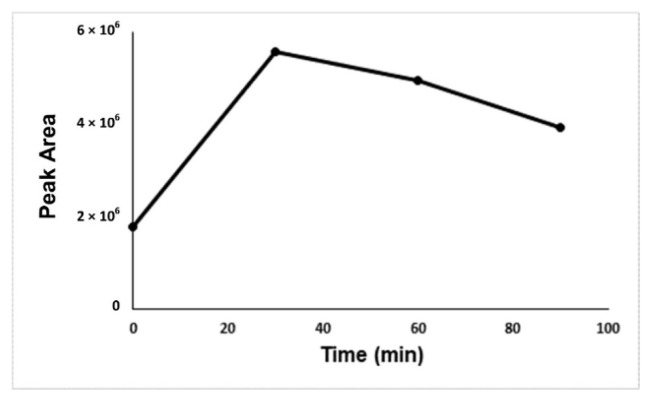
Optimization graph of incubation time against the formaldehyde-oxime peak area.

**Figure 3:**
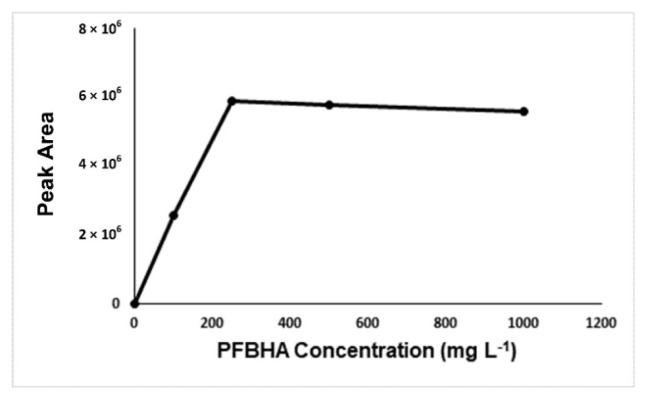
Effect of the derivatizing agent concentration on peak areas.

**Figure 4:**
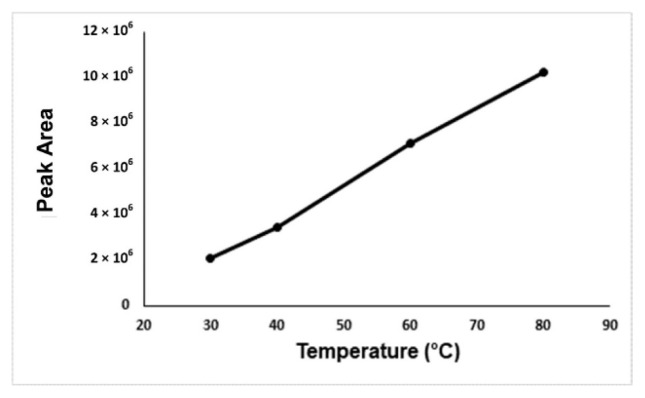
Optimization graph of the incubation temperature versus the peak areas.

**Figure 5:**
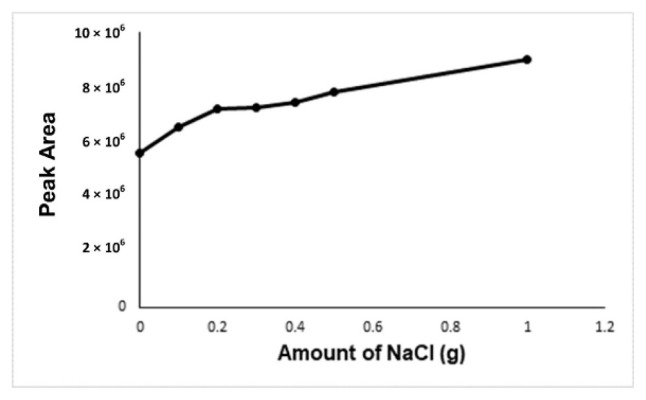
Effect of increasing NaCl concentration on the analyte signal.

For optimization of the developed method, derivatization time, derivatization temperature, concentration of derivatization reagent, salt effect, and the concentration of salt were investigated. Figures 2-5 show the optimum values for optimized parameters. The results showed that the optimum derivatization time was 30 min (Figure 2), and the concentration of derivatization reagent was 250 mg L^−1^ (Figure 3).

In the optimization of the derivatization temperature, the peak area of the formaldehyde-oxime increases with temperature. There could be two reasons for this. The first one is the positive effect of the increasing incubation temperature on the reaction efficiency, the other is that the solubility of the gases decreases with the temperature, so more analyte passes into the gas phase. As a result, it is seen that the increasing incubation temperature has a positive effect on the reaction efficiency. The highest peak area was obtained at an incubation temperature of 80 °C, but at this temperature, the vapor pressure of the water will increase too much and it will have a damaging effect on the column and the device. The 60 °C incubation temperature was accepted as the optimum temperature in terms of both giving enough signal and protecting the device and column (Figure 4). NaCl, KCl, and CaCl_2_ were carried out for the determination of the salt type effect on the analysis. Due to this, it was seen from the results no effect of salt type on the analysis, NaCl was chosen for the analysis. Because the increasing NaCl concentration has a positive effect on the analyte signal, 1.0 g of NaCl was determined as the optimal salt concentration. (Figure 5).

**Figure 6:**
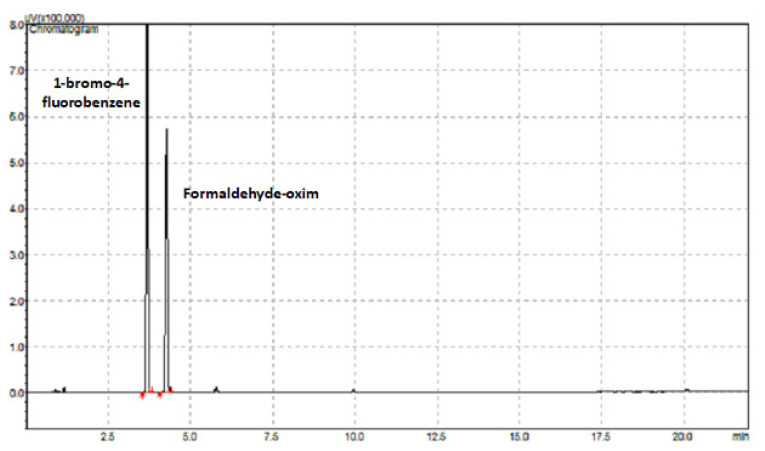
GC-FID chromatogram of 1-bromo-4-fluorobenzene and formaldehyde-oxime.

Figure 6 shows the chromatogram of the analysis of the internal standard 1-bromo-4-fluorobenzene (retention time: 3.681 min) and formaldehyde-oxime derivative (retention time: 4.269 min) at the determined optimum conditions.

Under the optimum conditions, formaldehyde solutions at concentrations ranging between 0.1–10 ppm gave a linear calibration curve:


$$\text{Peak area}=6.77\times 10^{4}\;\text{C}+4.59\times 10^{3}\;(\text{r}=0.9993)$$

For the developed method, the limit of detection (LOD) is 0.05 mg L^−1^ and limit of quantification (LOQ) is 0.167 mg L^−1^ (LOD = 3σ_bl_/m and LOQ= 10σ_bl_/m, where σ_bl_ denotes the standard deviation of a blank and m is the slope of the calibration curve). Three replicate analyses were made for each concentration and the relative standard deviation (RSD) of a certain set of readings varied in the range of 1.1%–5.4%, depending on the concentration. The coefficients of variation (CVs) of intra-assay and interassay measurements for the developed method were 5.4% and 7.9%, respectively (N = 5).

### 3.3. Analysis of real samples with the developed method

With the developed method, disperse dye, fluorescent pigment dye, and sodium naphthalene sulfoxylate formaldehyde, which are widely used in the textile industry and have the risk of containing formaldehyde, were analyzed. For the performed analysis, the results, dilution rates, and relative standard deviations (RSD %) are as in Table 1. The obtained results showed that the developed method could be applied for textile dyes and auxiliary chemicals successfully.

### 3.4. Statistical evaluation of the developed method

For the comparison method, formaldehyde samples were prepared according to the ISO 14184-1 method and were analyzed. The calibration was studied in the range of 0.1–3.75 mg L^−1^ concentration and the following calibration equation was obtained:


$$\text{Abs}=13.95\times 10^{-2}\;\text{C}-1.3\times 10^{-3}$$

For method validation, appropriate concentrations of sodium naphthalene sulfoxylate formaldehyde were carried out for both comparison and developed methods, and then the results were calculated with the dilution factors. The analysis was performed for N = 5 repetitive analyses. The compared methods showed no significant differences between the precision and accuracy of results (Table 2). The student t-test and F tests were used for statistical comparison of the population means and variances, respectively, at 95% confidence levels for both tests (Table 2).

## 4. Discussion

The ISO 14184-1 method, which is widely used for formaldehyde determination in textile quality control laboratories, is insufficient to meet the needs of the textile industry. One of the shortcomings of this method is that it is only applied to textile products (fabric, artificial leather, etc.). Due to the interfering effects of dyes and other auxiliary chemicals which were used in the textile industry on the spectrophotometric determination, these chemicals are not included in the scope of the method. Also, since this method measures formaldehyde in aqueous extracts of samples, it is not certain whether all of the formaldehyde on the fabric was measured. Another lack of the ISO14184-1 method is the relatively high limit of detection. Today, when the detection limits constantly decrease, the detection limit of 16 mg L^−1^ remains high compared to some textile standards, which limits the applicability of this method. Since the headspace sampling technique is used in the developed method, there is no need to perform the liquid-liquid extraction process used in gas chromatographic analysis. This also saves time and chemicals to be consumed. This successful sampling technique was combined with GC-FID to develop a new method for formaldehyde analysis. The developed method was successfully applied to different types of dyes and auxiliary chemicals used in the textile industry as a real example. In addition, the validation of the method was carried out by comparing it with the ISO 14184-1 method. The LOD value of the developed method can be used for the needed lower LOD of formaldehyde, which is increasingly important, in the future.

## Figures and Tables

**Table 1:** Real sample analysis with the aid of the developed method.

Analyte	Dilution Rate	Concentration (mg L^−1^)	RSD %
Disperse dye	100	367.8	0.13
Fluorescent pigment dye	100	4258.8	1.61
Sodium naphthalene sulfoxylate formaldehyde	200	712.6	0.11

**Table 2:** Statistical comparison of the developed method with ISO 14184-1 method for sodium naphthalene sulfoxylate formaldehyde with the aid of student t-test and F tests.

Method	Mean conc. (mg L^−1^)	SD (σ)	S^a^,^b^	t^a^,^b^	t_table_^b^	F^b^	F_table_^b^
Developed method	712.6	11.149	-	-	-	-	-
ISO 14184-1 method	703.6	8.258	9.81	1.42	2.306	1.23	6.39

a S^2^ = {(n_1_ – 1)s_1_^2^ + (n_2_ – 1)s_2_^2^} / (n_1_ + n_2_ – 2) and t = (ā_1_ – ā_2_)/{S(1 / n_1_ + 1 / n_2_)^1 / 2^}, where S is the pooled standard deviation, s_1_ and s_2_ are the standard deviations of the two populations with sample sizes of n_1_ and n_2_, and sample means of ā_1_ and ā_2,_ respectively (t has (n_1_ + n_2_ – 2) degrees of freedom); here, n_1_ = n_2_ = 5.

b Statistical comparison made on paired data produced with proposed and reference methods; the results given only on the row of the reference method.
